# Genetic analysis of protein efficiency and its association with performance and meat quality traits under a protein-restricted diet

**DOI:** 10.1186/s12711-023-00812-3

**Published:** 2023-06-02

**Authors:** Esther Oluwada Ewaoluwagbemiga, Giuseppe Bee, Claudia Kasper

**Affiliations:** 1 Animal GenoPhenomics, Agroscope, 1725 Posieux, Switzerland; 2 Department of Environmental Systems Science, ETH Zurich, Zurich, Switzerland; 3 Swine Research Unit, Agroscope, 1725 Posieux, Switzerland

## Abstract

**Background:**

An essential component in the development of sustainable pig production is the reduction of nitrogen excretion in fattening pigs. Pig feeds typically contain high levels of dietary crude protein, and due to incomplete conversion to muscle tissue, excess nitrogen is excreted, resulting in environmental problems such as nitrate pollution and greenhouse gas emissions. Therefore, improving protein efficiency (PE), i.e., the proportion of dietary protein that remains in the carcass, is desirable. The aim of this study was to estimate the heritability (h^2^) of PE and its genetic correlations with phosphorus efficiency, three performance, seven meat quality and two carcass quality traits when pigs were fed a 20% protein-restricted diet, using 1071 Swiss Large White pigs. To determine PE, the intake of feed with known nutrient content was accurately recorded for each pig and the nitrogen and phosphorus content of the carcass was determined using dual-energy X-ray absorptiometry.

**Results:**

We found an average PE of 0.39 ± 0.04 and a heritability of 0.54 ± 0.10. PE showed a high genetic correlation with phosphorus efficiency (0.61 ± 0.16), moderate genetic correlations with feed conversion ratio (− 0.55 ± 0.14) and average daily feed intake (− 0.53 ± 0.14), and a low genetic correlation with average daily gain (− 0.19 ± 0.19). While PE has favourable genetic correlations with the performance traits and some meat quality traits, there is a potentially unfavourable correlation of PE with meat colour (redness [r_g_ = − 0.27 ± 0.17]; yellowness [r_g_ = − 0.31 ± 0.18]) and intra-muscular fat (IMF; r_g_ = − 0.39 ± 0.15). Feed conversion ratio (FCR) also showed unfavourable genetic correlations with meat lightness, redness yellowness, IMF and cooking loss.

**Conclusions:**

PE is a heritable trait that can be considered in breeding programs to reduce the environmental impact of pig production. We found no strong negative correlation of PE with meat quality traits, and that there is potential to indirectly select for improved phosphorus efficiency. Selecting nutrient efficiencies might be a more suitable strategy to reduce nitrogen pollution from manure than focusing on FCR because the latter also shows genetic antagonism with some meat quality traits in our population.

**Supplementary Information:**

The online version contains supplementary material available at 10.1186/s12711-023-00812-3.

## Background

In the past, the focus of animal breeding has been on improving production traits, but today, sustainability concerns are increasingly gaining importance. An essential component to consider in the development of a sustainable pig production chain is the reduction of nitrogen excretion in fattening pigs. Compared to other crops, soybeans contain the highest amount of lysine, which is why soybean meal is often included in the feed to meet the lysine requirements of pigs, resulting in high dietary crude protein content in the feed [1]. Consequently, the excretion of excess nitrogen is increased in feces and urine, which contributes significantly to environmental problems such as nitrate pollution [2] and greenhouse gas emissions in the form of nitrous oxide when manure is applied to pastures and fields [3]. Besides pollution, the large-scale export of soybean from South America has caused massive deforestation in this region, loss of terrestrial biodiversity and deterioration of ecosystem services [4]. In addition, the increasing demand for meat, due to the rapid growth of the world population, has led to massive expansion of the land dedicated to soybean cultivation, thereby displacing natural vegetation and cultivation of other crops [5]. Lowering the crude protein content in pig feed could help reduce deforestation by lowering the demand for soybean, and therefore make it possible to use locally available plant protein sources in countries that import large amounts of soybean meal for pig feed. Therefore, considering the environmental and social impact of pig production, it is essential to improve protein efficiency (PE) in pigs. Protein efficiency is defined as the proportion of total dietary protein intake that is retained in the carcass. An improvement in PE would simultaneously decrease protein excretion, thereby reducing the contribution of pigs to environmental pollution and greenhouse gas emission. Since more than 50% of the ingested dietary protein in pigs is excreted as waste [6, 7], feed is an important factor to consider in improving PE. Pomar and Remus [9] reported that for every percent reduction in dietary nitrogen, nitrogen excretion could be decreased by 1.5%, thus improving PE. Ruiz-Ascacibar et al. [10], who investigated the influence of a 20% reduction in dietary protein on the PE of the Swiss Large White pig population, reported that pigs fed the 20% protein-reduced diet needed significantly more days on feed in the grower and finisher phase than those on the control diet. For instance, castrated males needed 2.5 more days in both the grower and finisher phases and females 5.9 and 8.4 more days in the grower and finisher phases, respectively [10]. However, in addition to nutritional strategies to improve PE, a genetic solution can be sought. Ruiz-Ascacibar et al. [9] reported that PE ranged from 37.5 to 55% within the protein-reduced treatment group. This variation between individuals to retain dietary protein can be exploited for the purpose of breeding. In addition, since excreted phosphorus also contributes to environmental pollution, it is interesting to investigate the relationship of PE with phosphorus efficiency for a possible indirect selection of the latter.

Feed conversion ratio (FCR) and residual feed intake (RFI) are the two most common traits considered for improving feed efficiency, and both are economically relevant. However, ecologically important traits such as PE should also be investigated to achieve a more sustainable pig production while maintaining the same production level. Although FCR and RFI are expected to be correlated with PE, selection for improved FCR and RFI would likely increase energy efficiency rather than PE [6, 7], since energy intake is a main factor driving feed intake [8]. Moreover, in poultry, selection for improved FCR and RFI with the aim of reducing nutrient excretion has been shown to be markedly less efficient than direct selection for the nitrogen or phosphorus excretion traits [11]. Nevertheless, in contrast to FCR and RFI, very few studies have investigated the possibility to improve PE, which can be due to several factors such as difficulties in phenotyping animals for this trait and the lack of approved and validated proxies.

Previous studies using a range of pig breeds, growth phases and diets reported heritabilities of 0.36 to 0.43 for nitrogen retention [12], 0.21 to 0.27 [13] and 0.36 to 0.42 [7] for PE. The nitrogen digestibility coefficient, which reflects the pig’s efficiency to digest dietary fiber and absorb proteins, had an estimated heritability of 0.27 to 0.56 [13]. For total nitrogen excretion during the finisher phase, Shirali et al. [15] reported a heritability [± standard error (SE)] of 0.32 (± 0.21). Finally, several quantitative trait loci (QTL) for nitrogen excretion traits were mapped and their effects and genetic architecture were described [16]. All these studies indicate a potential to select for protein-efficient pigs.

To estimate PE, the amount of protein ingested and the amount of protein retained in the animal need to be determined. While measuring the amount of protein ingested has been greatly facilitated with the use of automatic feeders that record daily feed intake of each pig, determining the amount of protein retention can be laborious, and previously-used methods are often either not suitable or too laborious and expensive for large-scale phenotyping. For instance, a direct, but expensive and laborious, method to determine PE is wet-chemistry analysis of the carcass and even of the whole body, including blood and organs [7]. Nitrogen retention can be predicted from lean meat content, which is estimated from the weights of primal cuts during dissection [12], which is laborious and subject to variation between butchers [17]. The deuterium dilution technique enables the estimation of nitrogen excretion traits from the empty body water content [15] and a nitrogen digestibility coefficient can be estimated from near-infrared spectroscopy of feces [14]. Lean meat content can also be estimated from a range of parameters, such as feed intake and growth patterns [13], or combinations of weight and backfat thickness [18, 19]. These indirect techniques often rely on specific assumptions and might not be generalized to different breeds or sexes, since specific prediction equations are required for each breed or sex [20]. Indirect methods also yield less precise estimates of lean mass (coefficient of determination R^2^ between 0.942 and 0.990 for predicting chemical values [18], and R^2^ of 0.896 and 0.908 for predicting lean mass from reference dissection [19]), even when specific estimation equations are applied. Here, we use a novel phenotyping strategy including a dual-energy X-ray absorptiometry (DXA) device in combination with automated feeders to estimate PE in a cheaper, more streamlined and faster, but still highly accurate way [21]. Carcass protein content can be estimated by DXA with high precision and accuracy (R^2^ between 0.986 and 0.998 [18]; R^2^ = 0.983 [21]). However, the accuracy for bone mineral content, which we used to compute phosphorus efficiency, is lower (R^2^ of 0.886 and 0.875 for empty body and carcass, respectively [21]; R^2^ between 0.816 and 0.851 [39] for predicting ash from DXA bone mineral content). DXA has been successfully applied to genetic studies of body composition in pigs [22] and other livestock species (reviewed in [20]). Moreover, in contrast to estimation methods that rely on point measurements, such as backfat thickness or loin muscle area, the information of total body as well as region-specific composition provided by DXA and other whole-body scan methods enable the targeted improvement of specific areas of the carcass through breeding programs.

Knowledge of the genetic correlations of PE with other traits of importance (such as meat quality and performance traits) is also important to account for possible trade-offs with traits included in current breeding programs. Studies have reported both favourable and unfavourable genetic relationships of the commonly-used feed efficiency traits (FCR and RFI) with meat quality traits and average daily gain [12, 23]. Phosphorus efficiency and its genetic correlation with PE is also an important trait to consider, as the low N:P ratio in pig feces results in excess phosphorus in soils when pig manure is used as a fertilizer [24, 25]. In addition, it is necessary to assess whether, at least on a phenotypic basis, PE could be associated with customers’ acceptance of meat based on flavor, tenderness and juiciness.

Thus, the major aim of this study was to estimate the heritability of PE and its genetic correlations with phosphorus efficiency, production, meat quality and carcass traits when dietary protein is reduced. In addition, we estimated the heritability of performance traits (ADG, ADFI and FCR) and meat quality and carcass traits, and their genetic correlations. Finally, we assessed differences in the sensory evaluation of meat between different PE groups based on tenderness, flavor and juiciness.

## Methods

### Animals and data sets

For the analysis of PE, we used 1071 pigs that originated from various datasets from previous experiments (294 pigs from [7]; 48 pigs from [26]; 48 pigs from a further study on protein and essential amino-acid reduction in the growth and finisher period or solely in the finisher period [27]; and an additional 681 pigs that were raised specifically for this study) (Table 1). In all the experiments explained below, pigs had ad libitum access to isocaloric diets that differed in crude protein or fiber content. Furthermore, in all the experiments, pigs were fed a grower diet from 20 to 60 kg body weight and a finisher diet from 60 kg to slaughter at 100 kg. In some experiments, pigs were kept until a 140-kg live BW and fed another specially formulated finisher diet from 100 to 140 kg [10]. In all the experiments, control diets were formulated according to the Swiss feeding recommendations for pigs.^1^ To determine the timing of transition between diets as well as the time of slaughter, all pigs were weighed individually each week. The data from previous experiments at Agroscope [28] included four experimental runs with eight farrowing series (i.e., batches of litters born in the same week), and pigs were assigned either to the control or protein-restricted diets. The protein-restricted diets contained 80% of the crude protein and digestible essential amino acids content of the respective control diets. The data collected by Bee et al. [26] included two experimental runs and pigs were assigned to three experimental treatments referred to as T95, T100, and T100-CF. Pigs in the T95 treatment were fed the control diets that complied with the BIOSUISSE regulation, which requires that 95% of the feed ingredients are of organic origin.^2^ The diets in the T100 treatment consisted of 100% feed ingredients that complied with the aforementioned regulation. The diet used in the T100-CF treatment was the same formulation as the T100, but with the crude fiber content being increased to 6% by including sunflower press cake, sainfoin and lupine. Pigs in this experiment were slaughtered at a BW of around 105 kg. The 48 pigs from Bee et al. [27] were assigned to three experimental treatments: C, the control diet, which was formulated using Swiss feeding recommendations in the grower and the finisher phase, RPF, in which the pigs were fed the control diet in the grower phase, but received a diet that contained 80% of the crude protein and digestible essential amino acids content of the control diet only in the finisher phase, and RPGF, a diet with a protein reduction in both the grower and finisher phases. Due to similarities between treatment groups in all the experiments described above, treatment groups were pooled over different experiments (Table 1). Pigs were slaughtered after they reached a 100-kg BW at individual weighing. Finally, the 681 pigs that were raised and phenotyped specifically for this study originated from 14 farrowing series (39 sires and 79 dams in total), and data were collected from October 2018 to June 2021. Forty-eight dams had one litter, 23 dams had two litters and eight dams had three litters. All sires and dams were of the Swiss Large White breed. All pigs in this experiment were fed a RPGF diet. Pigs in this experiment were slaughtered at an average body weight of 106 ± 5 kg. This was chosen to reflect current Swiss remuneration standards for carcasses, which penalize slaughter weights below 80 and above 100 kg with price deductions.

**Table 1:** Overview of the datasets with the differences in the sexes and carcass and dietary protein contents

Study	Sex			Sires	Dams	Carcass protein determination	T1	T2	Dietary raw protein (digestible lysine content (g/kg))^a^		
	Castrated male	Female	Entire male						Grower (20–60 kg BW)	Finisher I (60–100 kg BW)	Finisher II (100–140 kg BW)
Kasper et al. [7]^b^, [28]	92	92	110	17	56	Chemical analysis	Control	Control A	163 (9.72)	140 (7.80)	124 (7.15)
							RPGF	Treatment A	134 (7.88)	115 (6.36)	101 (5.76)
Bee et al.[26]^**c**^	24	24	–	3	9	DXA	T95	Control B	184 (11.04)	158 (8.49)	–
							T100	Control B	185 (10.96)	156 (8.71)	–
							T100-CF	Control C	185 (10.45)	156 (8.24)	–
Bee et al. [27]	24	24	–	4	9	DXA	Control	Control A	164 (10.07)	152 (8.13)	–
							RPF	Treatment B	164 (10.07)	121 (6.56)	–
							RPGF	Treatment A	132 (8.13)	121 (6.56)	–
Present study	329	352	–	39	79	DXA	RPGF	Treatment A	128 (7.80)	112 (6.06)	–
Total	469	492	110								

T1: name of the treatment group in the original study; RPGF: reduced protein diet in the grower and finisher stages; RPF: reduced protein diet in the finisher stage; T95: 95% of the feed ingredients were of organic origin; T100: 100% of the feed ingredients were of organic origin; T100-CF: 100% of the feed ingredients were of organic origin with 6% higher crude fiber content

T2: assigned name of treatment group in this study based on the quantity of dietary crude protein and crude fiber

^a^Energy content was the same (13.4 MJ) for both the grower and finisher diets and for all experimental treatments

^b^RP content in the study of Kasper et al. [7] was averaged over four series

^c^For the study of Bee et al. [26], all treatment groups had approximately the same quantity of crude protein, but T100-CF had a higher crude fiber content, hence, the different treatment group (Control C) assigned to T100-CF

Piglets were weaned at an average age of 27 ± 2 days after birth by removing the sow and were fed a standard starter diet with crude protein levels following the recommendation. When the pigs reached 22.3 (± 1.6) kg, they were placed in pens equipped with automatic feeders (single-spaced automatic feeder stations with individual pig recognition system by Schauer Maschinenfabrik GmbH & Co. KG, Prambachkirchen, Austria) and stayed on the starter diet. The experiment started at this stage and all pigs learned to access the automatic feeders, which allows monitoring of feed intake. The automatic feeder recorded all feeder visits and feed consumption per visit, from which the total feed intake of each pig was calculated [29]. The protein content of feed was monitored during production by near-infrared spectroscopy for each 500-kg batch. In addition, a sample was taken from each automatic feeder station each week and the crude protein content was determined by wet-chemistry methods. This was done to adjust for fluctuations in the crude protein content of raw materials when calculating PE, since the diet was formulated at the start of the study based on tabulated values of the ingredients. Every week, pigs were weighed individually, and, once the pig reached a live BW of 20 kg, it was allocated to a grower-finisher pen and the experimental treatments were started. This was done until a maximum number of 12 (or 24 or 48) pigs per pen (depending on the pen layout; minimum 1 m^2^ per pig and maximum 12 pigs/feeder) was reached. Pigs remained in the pen until slaughter.

Total and average daily feed (ADFI) was recorded, and average daily gain ($ADG$) and the feed conversion ratio ($FCR$) were calculated as:

$$ADG=\frac{live \, BW \, \left(kg\right) \, slaughter-live \, BW \, \left(kg\right) \, start}{age \, \left(days\right) \, slaughter-age \, \left(days\right) \, start},$$

$$FCR= \frac{ADFI}{ADG},$$

where $live \, BW \, \left(kg\right) \, slaughter$ and $age \, \left(days\right) \, slaughter$ are the live pre-slaughter body weight in kg and the age in days at slaughter, respectively, and $live \, BW \, \left(kg\right) \, start$ and $age \, \left(days\right) \, start$ are the exact body weight in kg and the age in days at the start of the grower phase, respectively.

### Protein and phosphorus efficiency

For the data in Kasper et al. [7], pigs were serially slaughtered at a body weight of 20 to 140 kg in 20-kg intervals and the nitrogen and phosphorus contents of the carcasses were determined by wet chemical analysis following the protocol described by [10]. For the other data sets, pigs were slaughtered at an average body weight of 106 ± 5 kg. Every week, each pig was weighed individually, and if it reached a BW of ~ 100 kg, it was slaughtered the week after. If a pig grew too slowly and did not reach the desired body weight in the same week as the last pigs in the series, it was slaughtered even if its body weight was below the desired weight. This was done for welfare reasons to avoid a pig remaining alone when its pen-mates had all been slaughtered. The left half of the carcass, including the whole head and the tail, was scanned with a Dual-energy x-ray absorptiometry (DXA; GE Lunar i-DXA, GE Medical Systems, Glattbrugg, Switzerland) to determine the lean tissue and bone mineral content. The lean tissue content and bone mass obtained from the DXA scans were used in the following prediction equation to estimate the protein content retained in the carcass [21].

$${CP}_{carcass} \left(g\right)=-482.745+0.23 \left({Lean}_{DXA}\left(\mathrm{g}\right)\times P\right),$$

$${Ph}_{carcass} \left(g\right)=-6.388+0.109 \left({BMC}_{DXA}\left(\mathrm{g}\right)\times P\right)+0.004 \left({Lean}_{DXA}\left(\mathrm{g}\right)\times P\right),$$

where ${CP}_{carcass} (g)$ is the crude protein content of the carcass in g, ${Lean}_{DXA}(\mathrm{g})$ is the lean meat content obtained with DXA in g, $P$ is the proportion of the weight of the left cold carcass-half weight (including the whole head and the tail) to the total cold carcass weight, ${Ph}_{carcass} \left(g\right)$ is the phosphorus content of the carcass in g, and ${BMC}_{DXA}(\mathrm{g})$ is the bone mineral content obtained with DXA in g. Protein and phosphorus efficiency of the carcass was thereafter calculated as the ratio of protein (or phosphorus) retained in the carcass (corrected for protein (or phosphorus) content in the carcass at a live body weight of around 20 kg, which is the start of the experiment and when feed intake was first monitored) to the total protein (or phosphorus) intake $({CP}_{feed} \, intake (\mathrm{g})$) during the experimental period.

$$protein \, efficiency= \frac{C{P}_{carcass} (g) slaughter-{CP}_{carcass} (g) start }{{CP}_{feed} \, intake (\mathrm{g})}.$$

The protein and phosphorus content ${CP}_{carcass} (g) start$ of the pigs at the start of this experiment was estimated from a sample of 38 piglets (12 females, 12 castrated males and 14 entire males). These 38 piglets were slaughtered at an average body weight of 20.98 ± 1.85 kg in a previous experiment and their carcass protein content was chemically determined [10]. The average protein content per kg carcass for each sex (female, entire male, and castrated male) was used to estimate ${CP}_{carcass} (g) start$ for pigs by multiplying the actual live body weight of pigs when they entered the experiment (i.e., at a body weight of ~ 20 kg) with the protein content per kg carcass of piglet, as previously determined from the 38 piglets [10].

### Meat quality traits and predictor variables in the study

In order to investigate possible trade-offs between PE and other traits of importance, such as meat quality and performance traits, additional traits were recorded on a subset of pigs that were raised specifically for this study (N = 509; Table 2). After exsanguination and evisceration, backfat thickness was measured at the 10th rib level on the left side of the hot carcass with a ruler. Thereafter, the eviscerated carcasses (left and right half) were weighed and then stored overnight at 4 °C. One day after slaughter, the *longissimus thoracii* (LT) muscle was excised (at the 10th to 12th rib level) from the left side of the cold carcass. The area of the LT was measured at the 11th to 12th rib level. A 1 × pixel JPEG image of the muscle was taken with a smartphone camera (always mounted on the same support structure to guarantee the same angle and distance to the object) together with a ruler for scale. Subsequently, the area was measured with the imageJ software (v1.53r). In addition, two 3-cm thick chops, labeled A and B, were cut. After a 20-min bloom period at 4 °C, L* (lightness), a* (redness), and b* (yellowness) values for chop A were measured using a spectrophotometer (model CM-2600d, Minolta, Dietikon, Switzerland). Subsequently, drip loss for the same chop was assessed as the quantity of purge generated during storage at 4 °C for 48 h. Thereafter, the samples were vacuum-sealed in plastic bags and cooked in a water bath at 72 °C for 45 min, then cooled in cold water for 15 min, rinsed to remove coagulated sarcoplasmic protein, dabbed dry and weighed to determine the cooking loss. The cooked chops were then stored at -20 °C until measuring the shear force. Shear force was determined on the cooked samples, which were slowly brought to ambient temperature. It was measured from five cores of each LT chop with a diameter of 1.27 cm perpendicular to the fiber direction using the Stable Micro System TA.XT2 Texture Analyzer (Godalming, Surry, UK) equipped with a 2.5-mm-thick Warner–Bratzler shear. The chops labeled B were freed from subcutaneous fat and then used to determine intramuscular fat content. The samples were placed in plastic bags, vacuumed-sealed and frozen at -20 °C until analysis. The frozen samples were lyophilized using a Delta 2–24 machine (Christ Delta 2–24, Kühner AG, Birsfelden, Switzerland) and the intramuscular fat content was determined by petrol ether extraction after acid hydrolysis (International Organization for standardization (ISO), 1999). Drip loss and cooking loss were calculated as:

$$\mathrm{Drip \, Loss }\left(\mathrm{DL}\right)=\frac{\mathrm{weight } \, 1-\mathrm{weight } \, 2}{\mathrm{weight } \, 1} \times 100,$$

$$\mathrm{Cooking \, Loss }\left(\mathrm{CL}\right)=\frac{\mathrm{weight } \, 2-\mathrm{weight } \, 3}{\mathrm{weight } \, 2} \times 100,$$

where $\mathrm{weight }1$ is the weight of the chop after a 20-min bloom period at 4 °C, $\mathrm{weight }2$ is the weight after 48 h at 4 °C, and $\mathrm{weight }3$ is the weight after cooking in a water bath at 72 °C for 45 min and cooled in cold water for 15 min with coagulated sarcoplasmic protein removed and dabbed dry. Predictor variables such as slaughter weight, slaughter age, treatment, year of change, sex and temperature were included in the genetic analysis of the efficiency traits, performance traits and meat quality traits. Slaughter weight is the weight of the animal at the time of slaughter and slaughter age is the age of the animal at slaughter. Due to the high correlation of slaughter age with slaughter weight (Pearson r = 0.77, *p* < 0.001), we used the residuals of a linear regression of slaughter age on slaughter weight (rAgeLW) in place of slaughter age to avoid collinearity. Year of change is the year when the animal entered into the experiment with an average body weight of 20 kg. Temperature is the average ambient temperature (± 3 days) in the room at the time when the animal entered the experiment.

**Table 2:** Traits measured as well protein and phosphorus efficiency and predictor variables used in analysis

Variable category	Trait/variable	Unit
Nutrient efficiency (N = 1071)	Protein efficiency	Proportion
	Phosphorus efficiency	Proportion
Meat quality (N = 510)	Meat colour (lightness, redness and yellowness)	International Commission on Illumination (CIE) color space coordinates
	Intra-muscular fat content	g/kg
	Water holding capacity (drip loss, cooking loss)	%
	Shear force	N
	Backfat thickness	mm
	Loin muscle area	cm^2^
Sensory evaluation of meat (N = 39)	Firmness	Rated by judges on a linear gradual scale ranging from 0.0 to 10.0
	Tenderness	Rated by judges on a scale 0–10
	Juiciness	Rated by judges on a scale 0–10
	Flavour	Rated by judges on a scale 0–10
Performance traits (N = 1071)	Average daily gain (ADG)	kg/d
	Feed conversion rate (FCR)	kg/kg
	Average daily feed intake (ADFI)	kg/d
Predictor variables (N = 1071)	Temperature	°C
	Sex	–
	Treatment	–
	Year of change	Calendar year
	Slaughter weight	kg
	Slaughter age (rAgeLW; residuals of slaughter age on slaughter weight)	days

### Sensory evaluation of meat

The objective of sensory evaluation was to identify potential differences in the perceived initial firmness (first bite), tenderness, juiciness and flavour of meat samples between three groups differing in PE (low, LPE; medium, MPE; and high, HPE). Two-cm thick chops were selected from pigs that were raised specifically for this study. Pigs with a PE lower than the mean PE-2σ were considered as LPE (group average PE of 0.35 $\pm$ 0.08), pigs with a PE higher than mean PE + 2σ were considered as HPE (group average PE of 0.57 $\pm$ 0.05), and pigs with a PE in between these two values were considered as MPE group (group average PE of 0.40 $\pm$ 0.01). In total, 12 chops (from 6 castrates and 6 females), 13 chops (from 6 castrates and 7 females), and 14 chops (from 6 castrates and 8 females) were used in the HPE, MPE, and LPE groups, respectively. Chops were taken between the 11th and 12th ribs (the same chop as that used for measuring loin muscle area) of the left half of the cold carcass 24 h postmortem and stored at − 80 °C. For the sensory analysis, chops were thawed for 24 h at 4 °C, blotted dry and stored at room temperature for approximately 1 h. Slices were grilled at 180 °C for 2.45 min on each side and cut into pieces of approximately 2 × 2 cm. The chops differed in the thickness of the attached fat (< 1.5 cm, 1.5 cm or 3 cm), which was statistically corrected in the model. The cut samples were kept at 60 °C until sensory testing. A panel of eight judges participated in the sensory tests. The intensity of the attributes of firmness, tenderness, juiciness, and total flavour was measured on an unstructured, gradual 10-cm line scale between low (0) and high (10) intensity. Prior to data collection, panelists participated in two training sessions to get familiar with the attributes of interest. In each of the six sessions, panelists evaluated two sets, each of them consisting of meat cuts of three different animals. Sample sets and samples within each test set were randomized according to a William Latin Square design. All samples were coded with a three-digit random number. Sensory data were collected using the software FIZZ (version 2.51 Biosystèmes, Couternon, France). White bread, still water and warm black tea were provided for neutralization of the mouth between samples. All tests were conducted at room temperature under daylight conditions in the sensory laboratory at Agroscope Posieux.

### Statistical analysis

All analyses were performed in the R software V 4.2.1 [30]. The *pedantics* package V 1.7 [31] was used to construct a multigenerational pedigree for the animal model and to derive pedigree-based parameters such as relatedness and inbreeding. The total pedigree contained 1468 unique individuals with a maximum pedigree depth of 12 generations, and from these, 682 were raised specifically for this study, 390 pigs were from previous experiments (described in Kasper et al. [7], as well as Bee et al. [26] and Bee et al. [27]), and 396 pigs with no phenotypes provided links for the individuals with PE data. All phenotyped individuals had both parents known. The mean inbreeding coefficient was equal to 0.00032. The genetic analysis (heritabilities and correlations) was performed using the variance and covariances obtained with the ASReml-R software [32]. To determine the effect of several variables on PE, first we ran a linear model that included all those variables and interactions as fixed effects. Interactions were included to account for the heterogeneity of data, i.e., in some experiments, entire males were used whereas in the others only females and castrated males were present, and some experiments had different dietary groups (Table 1). The structure of our data set was not fully cross-classified. Thus, as the results of the model for these interactions are not of interest per se, they are not discussed in this paper. Then, we selected the top model(s), i.e., all models within a delta AIC < 2 with the ‘dredge’ function in the MuMin package V 1.46.0 [34]. The variables retained in the top model were used as fixed effects in the univariate animal model to estimate the genetic and common environmental variance components (Table 3). The univariate animal models were performed using the following formula:

$$\mathbf{y}=\mathbf{X}\mathbf{b}+{\mathbf{Z}}_{\mathrm{a}}\mathbf{a}+{\mathbf{Z}}_{\mathrm{c}}\mathbf{c}+\mathbf{e},$$

where $\mathbf{y}$ is the vector of observations of the respective trait, $\mathbf{b}$ is the vector of fixed effects of the year the pig entered the experiment (year of change), slaughter weight, residuals of slaughter age on slaughter weight (rAgeLW), experimental treatment, sex, temperature, treatment × rAgeLW interaction, slaughter weight × sex interaction, treatment × sex interaction, and year of change × rAgeLW interaction. $\mathbf{X}$ is the incidence matrix relating records to fixed effects, $\mathbf{a}$ is the vector of random additive genetic effects, and $\mathbf{Z}$ is the corresponding incidence matrix. $\mathbf{c}$ is the vector of random litter effects, ${\mathbf{Z}}_{\mathrm{c}}$ is the corresponding incidence matrix, and $\mathbf{e}$ is the vector of random residual effects. Heritability was thereafter computed as the ratio of genetic variance to the phenotypic variance (${h}^{2}={V}_{A}/{V}_{P}$), where the phenotypic variance (${V}_{P}$) is the sum of the genetic variance (${V}_{A}$), litter (common environmental) effect (${V}_{CE}$) and residual variance (${V}_{R}$). The litter effect was calculated as the ratio of litter/common environment variance to phenotypic variance (${CE}^{2}={V}_{CE}/{V}_{P}$). The model distributions were visualized using the plot() method in ASReml-R.

**Table 3:** Description of the variables used in the models for efficiency, performance and meat quality traits

Variable	Included in the model		Levels	Variable type
	Univariate	Bivariate		
Year of change	Yes	Yes	9	Fixed categorical
Slaughter weight (kg)	Yes	Yes	1	Fixed continuous
Slaughter age (days)	Yes	Yes	1	Fixed continuous
Temperature (°C)	Yes	Yes	1	Fixed continuous
Treatment^a^	Yes	Yes	5	Fixed factor
Sex	Yes	Yes	3	Fixed factor
Animal	Yes	Yes	1071	Random factor
Sibship^b^	Yes	Yes	197	Random factor
Interactions^c^	Yes	–		
Treatment:slaughter age				
Year:slaughter age				
Slaughter weight:sex				
Treatment:sex				

^a^The variable ‘Treatment’ was used only in the univariate and bivariate model of efficiency and performance traits (PE, phosphorus efficiency, ADG, FCR, ADFI)

^b^The variable ‘Sibship’ was used only in the univariate and bivariate model of efficiency and performance traits (PE, phosphorus efficiency, ADG, FCR, ADFI)

^c^Interactions for meat quality traits differed depending on the top model selected for each meat quality trait

Genetic correlations were estimated using bivariate models that included the additive genetic and common environment covariance (the latter only for the correlations among the performance and efficiency traits), to which year, slaughter weight, treatment, sex, temperature, slaughter age (rAgeLW) were added as fixed effects (Table 3). An unconstrained variance/covariance matrix was assumed for the random models. For the bivariate analyses that included litter effect, we specified starting values by using the argument start.values = TRUE, which allows the model to change its random parameters. Genetic correlations were computed by rescaling additive genetic covariances by the variances.

While the data of the N = 1071 animals were used to estimate the heritability of PE and the performance traits and their genetic correlations, the heritability of meat quality was estimated on a subset of the animals (N = 510), for which these phenotypes were available. The univariate animal models used in the estimation of the heritability of the meat quality traits did not contain the fixed effect of treatment since the subset, for which meat quality measurements were available, had only one treatment group. The interactions between covariates identified in the selection step of the model differed between meat quality traits depending on the top model for each trait. A mixed-effects model in XLSTAT v 2021 was used for the phenotypic analysis of the sensory evaluation of meat to correct for the effect of session, judge, sex and fat thickness (Table 4), and Fisher’s least significance difference (LSD) mean separation test were performed for post-hoc analysis. The following model used was:

$${Y}_{ijk }=\mu +Session+J+Sex+PE+FT+{\varepsilon }_{ijk,}$$

where ${Y}_{ijk}$ are the observations for the dependent variables (initial firmness, tenderness, juiciness and flavour), $\mu$ is the overall mean, $Session$ is the random effect for session, $J$ is the random effect for judge, $Sex$ is the fixed effect of sex (castrates and females), $PE$ is the fixed effect of PE group (HPE, LPE and MPE), and $FT$ is the fixed effect of the thickness of fat adhering to chops.

**Table 4:** Description of the variables used in the model for the sensory analysis

Variable	Values	Number	Variable type
Judge		8	Random factor
Session		6	Random factor
Sex			Fixed factor
	Castrated males	18	
	Females	21	
Protein efficiency			Fixed factor
	HPE	12	
	LPE	14	
	MPE	13	
Fat thickness			Fixed factor
	< 1.5 cm	18	
	1.5 cm	12	
	3 cm	9	

*HPE* high protein-efficient, *LPE* low protein-efficient, *MPE* medium protein-efficient

## Results

### Estimates of heritability and litter effects

The descriptive statistics for the FCR (kg/kg), ADG (kg/day), and ADFI (kg/day) traits are in Table 5, and the descriptive statistics for the meat quality traits are in Table 6. Using the entire dataset (1071 pigs), PE, phosphorus efficiency, FCR, ADG and ADFI had a mean of 0.39 ± 0.04, 0.43 ± 0.05, 2.67 ± 0.23, 0.85 ± 0.11, and 2.26 ± 0.31, respectively (Table 5). Table 7 summarizes the estimates of the heritabilities and variance components of PE and of several performance and meat quality traits in Swiss Large White pigs. A heritability estimate ± SE of 0.54 ± 0.10 for PE was found in the carcass. The contribution of the litter effect to the phenotypic variance in carcasses for PE was almost zero (0.006 ± 0.03), which showed that growing up in the same early environment may not result in those pigs having more similar PE beyond the additive genetic effect. The heritability estimates of phosphorus efficiency, ADG, FCR, and ADFI were 0.27 ± 0.10, 0.45 ± 0.11, 0.39 ± 0.12, and 0.53 ± 0.12, respectively (Table 7). In addition, there was a litter effect of 0.06 ± 0.04, 0.15 ± 0.05, 0.11 ± 0.05 and 0.12 ± 0.05 for phosphorus efficiency, ADG, FCR and ADFI, respectively. The heritability estimates of meat quality and carcass traits were moderate to high ranging from 0.34 ± 0.12 for the LT area to 0.76 ± 0.12 for intramuscular fat (Table 7). In general, heritabilities could be estimated with high levels of confidence, as reflected in their low SE (Table 7).

**Table 5:** Descriptive statistics for the traits in each study and the overall dataset

Trait	Study^a^	*N*	Mean	SD
Protein efficiency	Bee et al. [26]	48	0.34	0.04
	Bee et al. [27]	48	0.39	0.04
	Present study	681	0.40	0.03
	Overall^b^	1071	0.39	0.04
Phosphorus efficiency	Bee et al. [26]	48	0.37	0.04
	Bee et al. [27]	48	0.40	0.03
	Present study	681	0.42	0.03
	Overall^b^	1071	0.43	0.05
FCR	Bee et al. [26]	48	2.54	0.15
	Bee et al. [27]	48	2.59	0.11
	Present study	681	2.76	0.14
	Overall^b^	1071	2.67	0.23
ADG	Bee et al. [26]	48	0.95	0.06
	Bee et al. [27]	48	0.94	0.07
	Present study	681	0.84	0.11
	Overall^b^	1071	0.85	0.11
ADFI	Bee et al. [26]	48	2.40	0.19
	Bee et al. [27]	48	2.44	0.18
	Present study	681	2.32	0.26
	Overall^b^	1071	2.26	0.31

^a^Descriptive statistics for the data of Kasper et al. [28] is reported in the study of Kasper et al. [7]

^b^Descriptive statistics for “Overall” also includes the data from Kasper et al. [7]

*N*—sample size

**Table 6:** Descriptive statistics for meat quality traits and meat sensory evaluation of meat protein efficiency groups

Trait	*N*	Mean	SD
*Meat quality*			
IMF	509	44.57	15.58
LMA	509	35.35	3.80
L*	509	51.82	2.28
a*	509	6.55	0.98
b*	509	3.91	0.89
Shear force	509	44.94	11.09
Drip loss	509	2.45	0.97
Cooking loss	509	26.91	2.67
Backfat thickness	509	21.17	5.60
*Sensory evaluation*			
HPE	12	0.57	0.05
MPE	13	0.40	0.01
LPE	14	0.35	0.08

*IMF* intramuscular fat, *LMA* Loin muscle area, L* meat lightness, a* meat redness, b* meat yellowness, *HPE* high protein-efficient, *MPE* medium protein-efficient, *LPE* low protein-efficient. *N* sample sizes.

**Table 7:** Variance, heritability and litter effect estimates for protein and phosphorus efficiency, performance, meat quality and carcass traits

Trait	N	σ^2^A	Heritability	σ^2^CE	Litter effect	σ^2^P
PE	1071	5.2 × 10^–4^ ± 1.2 × 10^–4^***	0.54 ± 0.10	5.8 × 10^–6^ ± 3.1 × 10^–5^	0.006 ± 0.03	9.6 × 10^–4^ ± 5.7 × 10^–5^
PhE	1071	5.6 × 10^–4^ ± 2.2 × 10^–4^**	0.27 ± 0.10	1.2 × 10^–4^ ± 8.5 × 10^–5^	0.06 ± 0.04	2.1 × 10^–3^ ± 1.1 × 10^–4^
ADG	1071	1.3 × 10^–3^ ± 3.7 × 10^–4^***	0.45 ± 0.11	4.2 × 10^–4^ ± 1.3 × 10^–4^***	0.15 ± 0.05	2.9 × 10^–3^ ± 1.8 × 10^–4^
FCR	1071	8.1 × 10^–3^ ± 2.7 × 10^–3^**	0.39 ± 0.12	2.2 × 10^–3^ ± 9.6 × 10^–4^*	0.11 ± 0.05	2.1 × 10^–2^ ± 1.2 × 10^–3^
ADFI	1071	1.2 × 10^–2^ ± 3.4 × 10^–3^***	0.53 ± 0.12	2.7 × 10^–3^ ± 1.1 × 10^–3^**	0.12 ± 0.05	2.3 × 10^–2^ ± 1.5 × 10^–3^
L*	509	1.60 ± 0.52 **	0.36 ± 0.10	–	–	4.43 ± 0.33
a*	509	0.50 ± 0.13 ***	0.58 ± 0.11	–	–	0.86 ± 0.07
b*	509	0.27 ± 0.08 ***	0.41 ± 0.10	–	–	0.65 ± 0.05
LMA	509	4.53 ± 1.75 **	0.34 ± 0.12	–	–	13.33 ± 1.13
IMF	509	154.09 ± 36.10 ***	0.76 ± 0.12	–	–	203.63 ± 19.00
ShF	509	35.37 ± 11.15 **	0.38 ± 0.10	–	–	92.47 ± 6.88
DL	509	0.62 ± 0.18 ***	0.40 ± 0.10	–	–	1.53 ± 0.11
CL	509	5.92 ± 1.88 **	0.36 ± 0.10	–	–	16.26 ± 1.19
BT	509	9.35 ± 2.41**	0.51 ± 0.10			18.37 ± 1.43

*PE* protein efficiency, *PhE* phosphorus efficiency, *ADG* average daily gain, *FCR* feed conversion ratio, *ADFI* average daily feed intake, L* meat lightness, a* meat redness, b* meat yellowness, *LMA* loin muscle area, *IMF* intramuscular fat, *ShF* shear force, *DL* drip loss, *CL* cooking loss, *BT* backfat thickness. *N* sample size *σ*^*2*^*A* additive genetic variance, *σ*^*2*^*CE* common environment variance, *σ*^*2*^*P* phenotypic variance

*** = P < 0.001, ** = P < 0.01, * = P < 0.05

### Genetic and phenotypic correlations

#### Efficiency and performance traits

Since PE is a new efficiency trait that is more in line with the goal of sustainability, we sought to assess potential trade-offs with the traits that are commonly included in breeding goals, such as FCR and other performance traits. The genetic correlations of PE with the performance traits were moderate and clearly different from zero. PE showed significant genetic correlations with phosphorus efficiency (0.61 ± 0.16), ADFI (− 0.53 ± 0.14), and FCR (− 0.55 ± 0.14), and a low genetic correlation with ADG (− 0.19 ± 0.19) (Table 8). The patterns of the phenotypic correlations of PE with phosphorus efficiency (0.53 ± 0.03) and performance traits, ADFI (− 0.36 ± 0.03), FCR (− 0.50 ± 0.03) and ADG (0.05 ± 0.04), were similar to those of the genetic correlations (Table 8). While the relationships of PE with phosphorus efficiency, ADFI and FCR are favourable (i.e., pigs with higher PE would be phosphorus-efficient, consume less feed and efficiently convert the feed), there seems to be an unfavourable relationship between PE and ADG, although with a corresponding high SE. FCR showed favourable genetic correlations of − 0.12 ± 0.21 and 0.57 ± 0.14 with ADG and ADFI, respectively (Table 8). Similarly, phenotypic correlations of FCR with ADG and ADFI were also favourable.

**Table 8:** Genetic (above the diagonal) and phenotypic correlations (below the diagonal) between protein efficiency and performance traits

	PE	PhE	ADG	FCR	ADFI
PE		0.61 ± 0.16	− 0.19 ± 0.19	− 0.55 ± 0.14	− 0.53 ± 0.14
PhE	0.53 ± 0.03		− 0.21 ± 0.25	− 0.15 ± 0.24	− 0.25 ± 0.21
ADG	0.05 ± 0.04	− 0.003 ± 0.04		− 0.12 ± 0.21	0.74 ± 0.10
FCR	− 0.50 ± 0.03	− 0.31 ± 0.03	− 0.38 ± 0.03		0.57 ± 0.14
ADFI	− 0.36 ± 0.03	− 0.27 ± 0.04	0.59 ± 0.03	0.50 ± 0.03	

*PE* protein efficiency, *PhE* phosphorus efficiency, *ADG* average daily gain, *FCR* feed conversion ratio, *ADFI* average daily feed intake

#### Efficiency, meat quality and carcass traits

PE showed favourable genetic correlations with LMA (0.72 ± 0.16) and backfat thickness (− 0.37 ± 0.16) (Table 9). However, PE may have potentially unfavourable genetic correlations with meat redness (a*), meat yellowness (b*), IMF and cooking loss, although the SE for these correlations were quite high compared to the estimates in Table 9. A similar pattern was also observed with the phenotypic correlations of PE with meat quality traits (Table 9). FCR showed unfavourable genetic correlations with meat colour (L* = − 0.34 ± 0.18, a* = 0.46 ± 0.16, and b* = 0.35 ± 0.19), IMF (0.28 ± 0.17) and cooking loss (− 0.47 ± 0.17) (Table 10). The same pattern holds for the phenotypic correlations of FCR with meat colour, IMF and cooking loss.

**Table 9:** Genetic (above the diagonal) and phenotypic correlations (below the diagonal) between protein efficiency and meat quality traits

	PE	L*	a*	b*	LMA	IMF	ShF	DL	CL	BT
PE		0.06 ± 0.20	− 0.27 ± 0.17	− 0.31 ± 0.18	0.72 ± 0.16	− 0.39 ± 0.15	0.003 ± 0.20	0.22 ± 0,19	0.59 ± 0.15	− 0.37 ± 0.16
L*	0.03 ± 0.05		− 0.16 ± 0.19	0.27 ± 0.19	0.36 ± 0.24	0.24 ± 0.18	− 0.57 ± 0.14	0.17 ± 0.22	0.19 ± 0.21	0.05 ± 0.20
a*	− 0.11 ± 0.06	0.15 ± 0.06		0.84 ± 0.06	− 0.33 ± 0.21	0.42 ± 0.14	− 0.39 ± 0.17	− 0.12 ± 0.19	− 0.50 ± 0.16	− 0.25 ± 0.18
b*	− 0.12 ± 0.05	0.54 ± 0.04	0.76 ± 0.02		− 0.28 ± 0.23	0.58 ± 0.13	− 0.61 ± 0.14	− 0.12 ± 0.20	− 0.43 ± 0,18	− 0.09 ± 0.19
LMA	0.22 ± 0.06	0.05 ± 0.06	− 0.19 ± 0.06	− 0.12 ± 0.06		− 0.04 ± 0.21	− 0.09 ± 0.25	0.51 ± 0.20	0.93 ± 0.10	0.14 ± 0.23
IMF	− 0.18 ± 0.06	0.26 ± 0.0	0.25 ± 0.06	0.35 ± 0.05	− 0.14 ± 0.06		− 0.18 ± 0.18	− 0.32 ± 0.17	− 0.20 ± 0.18	0.14 ± 0.17
ShF	0.01 ± 0.05	− 0.59 ± 0.03	− 0.34 ± 0.05	− 0.51 ± 0.04	0.03 ± 0.06	− 0.18 ± 0.05		− 0.09 ± 0.22	0.08 ± 0.21	0.11 ± 0.20
DL	0.14 ± 0.05	0.03 ± 0.05	− 0.02 ± 0.05	− 0.04 ± 0.05	0.24 ± 0.06	− 0.17 ± 0.06	0.01 ± 0.05		0.39 ± 0.18	− 0.11 ± 0.20
CL	0.17 ± 0.05	− 0.07 ± 0.05	− 0.20 ± 0.05	− 0.17 ± 0.05	0.43 ± 0.05	− 0.17 ± 0.05	0.20 ± 0.05	0.39 ± 0.04		0.23 ± 0.19
BT	− 0.18 ± 0.05	0.07 ± 0.05	0.03 ± 0.06	0.08 ± 0.05	0.06 ± 0.06	0.12 ± 0.06	− 0.10 ± 0.05	− 0.01 ± 0.05	− 0.04 ± 0.05	

*PE* protein efficiency, L* meat lightness, a* meat redness, b* meat yellowness, *LMA* loin muscle area, *IMF* intramuscular fat, *ShF* shear force, *DL* drip loss, *CL* cooking loss, *BT* backfat thickness

**Table 10:** Estimates of genetic and phenotypic correlations between performance traits and meat quality and carcass traits

	Genetic correlations								
	L*	a*	b*	LMA	IMF	ShF	DL	CL	BT
ADG	0.04 ± 0.19	0.11 ± 0.17	0.17 ± 0.18	− 0.18 ± 0.22	0.16 ± 0.16	− 0.09 ± 0.19	− 0.02 ± 0.19	− 0.04 ± 0.19	0.33 ± 0.16
FCR	− 0.34 ± 0.18	0.46 ± 0.16	0.35 ± 0.19	− 0.67 ± 0.18	0.28 ± 0.17	0.01 ± 0.20	− 0.06 ± 0.20	− 0.47 ± 0.17	0.22 ± 0.18
ADFI	− 0.13 ± 0.19	0.33 ± 0.15	0.33 ± 0.17	− 0.53 ± 0.17	0.30 ± 0.15	− 0.07 ± 0.19	− 0.001 ± 0.19	− 0.30 ± 0.17	0.42 ± 0.14

	Phenotypic correlations								
	L*	a*	b*	LMA	IMF	ShF	DL	CL	BT
ADG	0.05 ± 0.06	0.13 ± 0.06	0.13 ± 0.06	− 0.12 ± 0.06	0.17 ± 0.06	− 0.05 ± 0.06	− 0.002 ± 0.06	− 0.08 ± 0.06	0.25 ± 0.06
FCR	− 0.15 ± 0.05	0.10 ± 0.06	0.04 ± 0.05	− 0.14 ± 0.06	0.11 ± 0.06	0.03 ± 0.05	− 0.19 ± 0.05	− 0.17 ± 0.05	0.10 ± 0.05
ADFI	− 0.04 ± 0.06	0.19 ± 0.06	0.15 ± 0.06	− 0.21 ± 0.06	0.25 ± 0.06	− 0.05 ± 0.06	− 0.12 ± 0.06	− 0.20 ± 0.06	0.31 ± 0.05

*ADG* average daily gain, *FCR* feed conversion ratio, *ADFI* average daily feed intake, L* meat lightness, a* meat redness, b* meat yellowness, *LMA* loin muscle area, *IMF* intramuscular fat, *ShF* shear force, *DL* drip loss, *CL* cooking loss, *BT* backfat thickness

### Sensory evaluation of meat

The results of the sensory evaluation of meat showed a significant influence of judge and session on all the sensory attributes (P < 0.01), except for the influence of session on juiciness. A significant influence of PE group on juiciness was observed (P < 0.01). However, MPE and LPE were not significantly different for juiciness, but a significant difference was observed between LPE and HPE as well as between MPE and HPE. This probably means that the judges graded the juiciness of LPE and MPE meat samples equally, while they graded HPE samples as less juicy (P < 0.05). PE group did not show a significant influence on initial firmness, tenderness and flavor (P > 0.05), suggesting that an improvement in PE may not influence the sensory attributes considered in this study except for juiciness. However, our results should be followed-up with a consumer panel to investigate whether consumers perceive the meat of the distinct PE groups as different.

## Discussion

In this study, we estimated the heritability of PE, phosphorus efficiency and a range of performance, meat and carcass quality traits and their genetic correlations in a population of Swiss Large White pigs under various levels of dietary protein and amino acid supply. As already suggested previously [7], our results clearly show a major potential for selecting protein efficient pigs, mostly without any apparent expected negative impact on production, meat and carcass quality traits.

### Protein and phosphorus efficiency

The heritability estimate for protein efficiency reported in this study was higher than that reported previously for the same population [7], where carcass protein was determined by wet chemistry, a very accurate method. This may be due to the larger size of the sample used in the current study, to which the previous data were integrated. However, it is more likely that it results from the use of different statistical models (MCMCglmm). Applying the model of Kasper et al. [7] to the data of this study resulted in a highly similar heritability estimate of 0.38 [0.23, 0.53] (see Additional file 1: Fig. S1). Although only a few studies have investigated PE in pigs, some have probed similar nutrient efficiency traits, such as total nitrogen excretion and nitrogen digestibility coefficient, and have reported varying heritabilities depending on the breed, growth phase, and diet [11–14]. Our study showed a higher heritability for PE compared to most of the previous studies. These differences could be due to differences in breed and diet, but also to differences in the methods used to estimate nutrient efficiency, which, in our case, might be more reliable compared to the use of indirect estimation methods, such as the deuterium technique or the estimation from lean meat content from dissections or production parameters. Whole-body scans might yield higher heritability estimates of body composition and thus of PE than deriving body composition from point measurements such as backfat thickness by ultrasound, due to the reduction of error.

Consistent with previous work, our results confirm that nutrient efficiency traits are heritable and can be harnessed to substantially reduce environmental pollution in pig production. However, in the practical implementation of breeding, it should be kept in mind that PE as a ratio trait may have a disadvantage. As argued by Zetouni et al. [35], direct selection for a ratio trait such as PE may not lead to the desired result, as it is not certain whether the improvement comes from the counter trait (e.g., protein retention), the denominator trait (e.g., protein intake), or both. Therefore, it is advisable to use a multi-trait selection approach (i.e., selecting for both protein retention and protein intake) to achieve the highest genetic gain for a ratio trait [35]. In addition to direct genetic effects, the composition of the gut microbiome has been shown to influence general feed efficiency [36] and the genetic make-up of the host together with the composition of the microbiome explain more of the phenotypic variation in digestive efficiency of nitrogen than additive genetic variation alone [14]. Thus, this important source of phenotypic variation should be considered in breeding programs.

### Performance traits

This study reports an influence of slaughter weight, sex and treatment group on average daily gain (ADG), feed conversion ratio (FCR) and average daily feed intake (ADFI), which is similar to the results of Kasper et al. [7] and Ruiz-Ascacibar et al. [10]. The heritabilities for performance traits were also mostly in line with those of other studies. We found moderate heritabilities for ADG and ADFI, with that for ADG being lower than that reported by Shirali et al. [15] (h^2^ = 0.64 ± 0.19), but higher than the estimates reported by Verschuren [13] (h^2^ = 0.27 and h^2^ = 0.43 for ADG and ADFI, respectively) and Kavlak and Uimari [37] (h^2^ = 0.25 ± 0.06 for ADG). FCR was also clearly heritable in the present study, i.e. h^2^ = 0.39 ± 0.12, which is similar to, although slightly higher than, the estimates reported by Saintilan et al. [12]. In contrast, Shirali et al. [15] reported a lower heritability of 0.26 ± 0.20 over the 60 to 140 kg body weight period. Previously, we also reported very low heritability estimates for FCR and ADG [7]. These differences in estimates between the studies could be due to the larger size of the sample used here, that included 777 additional individuals. The heritability estimate for FCR may differ depending on growth phase and test period (e.g., at only the grower or finisher phase or throughout all growth phases) as reported by some studies where multiple QTL for ADG and for FCR at different growth phases [16, 38, 39] were identified. Our moderate heritability estimates for meat quality traits are similar to those of previous studies [15, 33, 40, 41]. As in our study, redness of the meat consistently showed higher heritability estimates than lightness and yellowness of the meat.

### Genetic and phenotypic correlations with performance traits

Favourable genetic correlations were observed between PE and phosphorus efficiency, FCR and ADFI. These results show that selection for increased PE would result in pigs that are also more phosphorus-efficient, consume less feed and efficiently convert the feed into body (and, in particular, muscle) mass. We observed a low negative relationship between PE and ADG, which indicates that selection for increased PE might influence the daily growth of pigs. Similar relationships were found in the work of Déru et al. [14] on nitrogen digestible coefficient and of Verschuren [13] on PE. In the latter study, the genetic relationships of PE with ADG, FCR and ADFI became stronger with increasing pig age. This suggests that the genetic factors that underlie these traits and their covariances may vary across growth phases. Considering the ecological footprint and feed costs, a focus on the finishing phase would therefore be more relevant, as most feed is consumed in this period. A possible unfavourable relationship between PE and ADG may be due to an indirect effect of lower feed intake since there is a negative relationship between PE and ADFI. However, the relationship between PE and ADG may not be linear, i.e., protein-efficient pigs do not necessarily grow more slowly during each growth phase. For example, Verschuren [13] reported a low positive, i.e., favourable, genetic correlation (r_G_ = 0.11) of PE with ADG in the starter phase, but a low negative (r_G_ = − 0.11) in the grower phase as well as a stronger, unfavourable genetic correlation (r_G_ = − 0.43) in the finisher phase. Furthermore, Shirali et al. [16] also found QTL for nitrogen excretion to be unfavourably associated with ADG only during the 90 to 120 kg body weight growth phase. Saintilan et al. [12] also reported favourable genetic correlations of nitrogen excretion efficiency with ADG, FCR and ADFI, but that with FCR was close to 1, in contrast to the r_G_ of − 0.55 ± 0.14 in our study. The reason for some of these discrepancies observed between our study and that of Saintilan et al. [12] may be due to differences in the methodology used, the traits, and sample size, but most importantly probably to the mixing of growth phases.

### Genetic and phenotypic correlations with meat and carcass quality traits

In this study, while PE did not show significant correlations with meat lightness (L*) and shear force (i.e., these traits are most likely not influenced by PE), we found favourable genetic correlations of PE with LMA and backfat thickness (i.e., an increase in PE will increase loin muscle area and decrease backfat thickness). Considering the high genetic correlation of PE with LMA, the latter could be considered for predicting PE, but this requires the slaughter of the animal. Because of the intermediate negative correlation of BT with PE, BT is rather less interesting as a proxy for PE, since a considerable loss of genetic progress in PE would be expected. The same applies to dressing percentage, which also had a rather low genetic correlation with PE (rG = 0.28 ± 0.19; [see Additional file 2: Table S1]). PE showed an unfavourable genetic correlation with meat colour (redness and yellowness), IMF and cooking loss. However, the genetic correlations of PE with meat redness and yellowness were moderate, but clearly different from zero. This indicates that genetic improvement of PE might reduce the redness and yellowness of the meat (the meat might look paler), which could be perceived as unattractive by consumers. However, this may depend on the starting value of the meat colour. Déru et al. [14] reported non-significant genetic correlations of nitrogen digestible coefficient with meat lightness, redness and yellowness, which were in an unfavourable direction for meat lightness and redness depending on the type of diet (conventional or high fiber diet). Additional experiments, such as the visual assessment and preferences of meat colour by consumers, should be conducted to investigate whether differences in meat colour within the expected range would lead to different consumer decisions. The sensory analysis in our study suggests that there are no apparent conflicts between PE and the way the judges perceived the initial firmness, tenderness and flavor of the meat of a small subset of samples. However, the trained judges perceived the meat from highly protein-efficient pigs as less juicy than that from pigs that had average or below-average PE, and a test with a consumer panel should be conducted to investigate whether this would affect the consumers’ buying decision.

The genetic correlations of FCR also showed possible unfavourable relationships with meat lightness, meat redness, meat yellowness, IMF and cooking loss, which suggest that an indirect selection for higher PE by selecting for lower FCR in Swiss Large White pigs would not only result in lower genetic gain due to the moderate genetic correlation between PE and FCR, but also lead to lighter meat. This agrees with the study of Saintilan et al. [12] who reported significant negative genetic correlations of FCR with meat lightness for the Large White breed.

## Conclusions

Our results clearly show that PE is heritable, and breeding for protein-efficient pigs is possible. It should be noted that, in this study, the genetic parameters of PE were estimated predominantly under conditions of low dietary protein availability. Together with the phenotypic results of the influence of diet presented in this work, and the still unresolved question of genotype-by-diet interactions, this shows the importance of the dietary environment, and the influence of dietary protein content should be considered accordingly. There seems to be a potential for indirectly selecting improved phosphorus efficiency due to its high genetic correlation with PE. Selection for PE does not appear to have major conflicts with meat quality and carcass traits, except for meat redness and yellowness, IMF and cooking loss, which may need to be closely monitored. Regarding the production traits, genetic correlations are favourable, although average daily gain should be monitored to avoid slower growth with increased PE. To achieve a significant reduction in pollutant levels in manure, selection on conventional efficiency traits alone, such as FCR or RFI, might not be sufficient. Furthermore, we found that FCR shows a genetic antagonism with meat quality traits in the population studied. Thus, including PE in the breeding goals would better contribute to a sustainable and environmentally friendly pig production.

## Supplementary Information

**Additional file 1: Figure S1.** Heritability estimate (h^2^), common environment effect (CE^2^; ratio of the variance of litter to the phenotypic variance) and residual variance (r^2^; the ratio of the residual variance to the phenotypic variance) of protein efficiency (A) using MCMCglmm. Posterior distributions of the respective variance components (upper part), points representing single estimates are shown together with a box plot (with median; whiskers represent the 95% credible interval).

**Additional file 2: Table S1.** Genetic correlations (above the diagonal) and phenotypic correlation (below the diagonal) of dressing percentage with protein efficiency [42].

## Data Availability

The data that support the findings of this study and the code used for models and the statistical analysis will be made publicly available in Zenodo (Doi: 10.5281/zenodo.6985500) after an embargo period of two years.

## Footnotes

1 Fütterungsempfehlungen und Nährwerttabellen für Schweine (Feeding recommendations and nutrient tables for pigs). Agroscope, Posieux, Switzerland. Retrieved on 31 January 2017 from https://www.agroscope.admin.ch/agroscope/fr/home/services/soutien/aliments-pour-animaux/apports-alimentaires-recommandes-pour-les-porcs.html.

2 Bio-Suisse: Production standards (https://international.bio-suisse.ch/en/import-with-bio-suisse/documents-and-downloads.html), retrieved on March 15, 2023.
