# Supplementary material for: Genetic analysis of protein efficiency and its association with performance and meat quality traits under a protein-restricted diet
Source: Genet Sel Evol. 2023 Jun 2;55:35. doi: 10.1186/s12711-023-00812-3 (PMC10236592; doi:10.1186/s12711-023-00812-3)
Supplement: Supplementary file 1 — Additional file 1: Figure S1. Heritability estimate (h2), common environment effect (CE2; ratio of the variance of litter to the phenotypic variance) and residual variance (r2; the ratio of the residual variance to the phenotypic variance) of protein efficiency (A) using MCMCglmm. Posterior distributions of the respective variance components (upper part), points representing single estimates are shown together with a box plot (with median; whiskers represent the 95% credible interval). [file 12711_2023_812_MOESM1_ESM.docx]

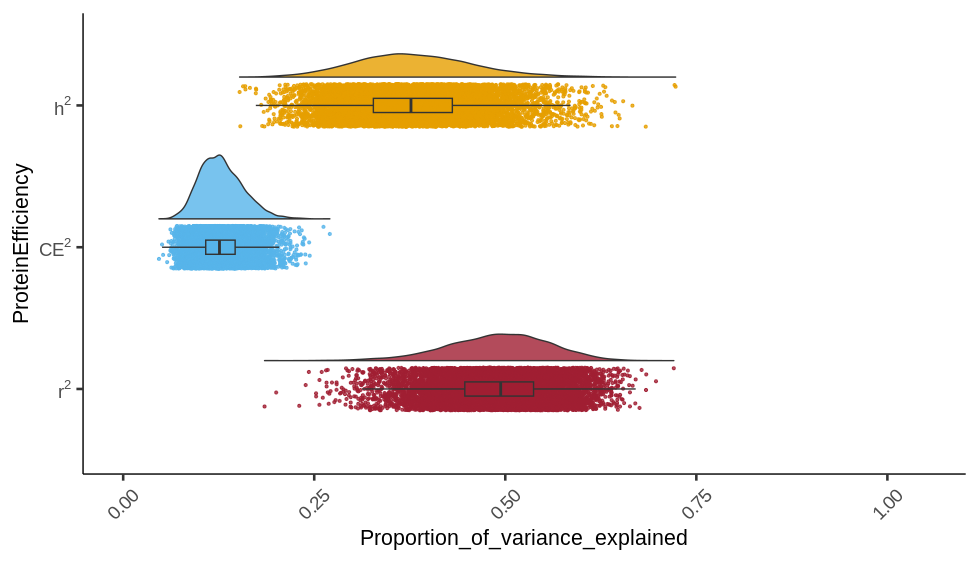


Proportion of variance explained

**Figure S1** Heritability (h^2^), common environment effect (CE^2^; the ratio of the variance of litter to the phenotypic variance) and residual variance (r^2^; the ratio of the residual variance to the phenotypic variance) of protein efficiency using the R package MCMCglmm. Posterior distributions of the respective variance components (upper part), points representing single estimates are shown together with a box plot (with median; whiskers represent the 95% credible interval).
