# Supplementary material for: Genetic analysis of protein efficiency and its association with performance and meat quality traits under a protein-restricted diet
Source: Genet Sel Evol. 2023 Jun 2;55:35. doi: 10.1186/s12711-023-00812-3 (PMC10236592; doi:10.1186/s12711-023-00812-3)
Supplement: Supplementary file 2 — Additional file 2: Table S1. Genetic correlations (above the diagonal) and phenotypic correlation (below the diagonal) of dressing percentage with protein efficiency [42]. [file 12711_2023_812_MOESM2_ESM.docx]

**Table S1** Genetic correlations (above diagonal) and phenotypic correlation (lower diagonal) of dressing percentage with protein efficiency

|  | **Protein efficiency** | **Warm dressing percentage** | **Cold dressing percentage** |
| --- | --- | --- | --- |
| Protein efficiency |  | 0.28 ± 0.19 | 0.31 ± 0.18 |
| Warm dressing percentage | 0.14 ± 0.05 |  | 0.99 ± 0.01 |
| Cold dressing percentage | 0.16 ± 0.05 | 0.91 ± 0.01 |  |
